# Artificial Intelligence in Obsessive-Compulsive Disorder: A Systematic Review

**DOI:** 10.1007/s40501-025-00359-8

**Published:** 2025-06-14

**Authors:** Jiyeong Kim, Juan Pablo Gonzalez Pacheco, Ashleigh Golden, Elias Aboujaoude, Peter van Roessel, Aayushi Gandhi, Pavithra Mukunda, Tatevik Avanesyan, Haopeng Xue, Ehsan Adeli, Jane Paik Kim, Manish Saggar, Shannon Wiltsey Stirman, Eric Kuhn, Kaustubh Supekar, Kilian M. Pohl, Carolyn I. Rodriguez

**Affiliations:** 1 Stanford Center for Digital Health, Stanford School of Medicine, Stanford, CA USA; 2 Department of Psychiatry and Behavioral Sciences, Stanford School of Medicine, Stanford, CA USA; 3 Artificial Intelligence for Mental Health Initiative (AI4MH), Department of Psychiatry and Behavioral Sciences, Stanford University, Stanford, CA USA; 4 Department of Computer Science, Stanford University, Stanford, CA USA; 5 Department of Biomedical Data Science, Stanford School of Medicine, Stanford, CA USA; 6 Dissemination and Training Division, National Center for PTSD, VA Palo Alto Health Care System, Palo Alto, CA USA; 7 Veterans Affairs Palo Alto Health Care System, Palo Alto, CA USA; 8 Department of Electrical Engineering, Stanford University, Stanford, CA USA; 9 Program in Internet, Health and Society, Cedars-Sinai Medical Center, Los Angeles, CA USA

**Keywords:** AI, OCD, Obsessive-compulsive disorder, Mental health, Diagnosis, Treatment

## Abstract

**Purpose of Review:**

Obsessive-compulsive disorder (OCD) is a chronic and disabling condition, often leading to significant functional impairments. Despite its early onset, there is an average delay of 17 years from symptom onset to diagnosis and treatment, resulting in poorer outcomes. This systematic review aims to synthesize current findings on the application of AI in OCD, highlighting opportunities for early symptom detection, scalable therapy training, clinical decision support, novel therapeutics, computer vision-based approaches, and multimodal biomarker discovery.

**Recent Findings:**

While previous reviews focused on biomarker-based OCD detection and treatment using machine learning (ML), the findings of the current review add information about novel applications of deep learning technology, specifically generative artificial intelligence (GenAI) and natural language processing (NLP). Among the included 13 articles, most studies (84.6%) utilized secondary data analyses, primarily through GenAI/NLP. Nearly 77% of these studies were published in the past two years, with high quality of evidence. The primary focus areas were enhancing treatment and management, and timely OCD detection (both 38.5%); followed by AI tool development for broader mental health applications.

**Summary:**

AI technologies offer transformative potential for improvements related to OCD if diagnosis occurs earlier after onset; thereby lessening the consequential economic burden. Prioritizing investment in ethically sound AI research could significantly improve OCD outcomes in mental health care.

**Supplementary Information:**

The online version contains supplementary material available at 10.1007/s40501-025-00359-8.

## Introduction

Obsessive-compulsive disorder (OCD) is a chronic disorder characterized by intrusive, unwanted thoughts (obsessions) and repetitive behaviors (compulsions) performed to reduce the distress provoked by thoughts [1]. OCD is common and disabling, with a lifetime prevalence of 2% and significant functional impairments impacting work, relationships, and quality of life [2, 3]. OCD starts early in life, with an average age of symptom onset of 19.5 years [3]; however, symptoms often go undetected and undiagnosed. On average, there is a delay of 17 years from the onset of OCD symptoms until diagnosis and engagement with evidence-based treatment [4]. At the same time, a longer duration of untreated illness leads to poorer prognosis and outcomes [5, 6].

The American Psychiatric Association clinical practice guidelines recommend cognitive behavioral therapy (CBT) with exposure and response prevention (ERP) and pharmacotherapy with a serotonin reuptake inhibitor (SRI) as first-line treatments for OCD [2]. ERP involves patients voluntarily exposing themselves to a feared object or idea while refraining from their compulsive behaviors [7]. Although ERP is effective, availability is limited [8, 9] due to the lack of ERP-trained providers in the mental health care workforce [10]. Many patients respond to treatment with SRIs; however, few individuals achieve minimal symptoms from SRIs alone, and side effects can be impairing [2]. Improving access to effective psychotherapies and identifying more effective pharmacological treatments with fewer side effects would constitute a major advance.

Artificial Intelligence (AI) algorithms aim to perform tasks at the level of human intelligence [11]. For example, the AI subfield of Natural language processing (NLP) is developing machine and deep learning algorithms to interpret human language and is increasingly being utilized model in mental health research to analyze large datasets of clinical notes, neuroimaging, social media posts, and patient-reported outcomes [12]. Although AI holds promise for transformative change in mental health care, it is not a remedy for every challenge, and careful attention and study is needed to understand both the opportunities and pitfalls [13, 14]. Safe and successful integration of AI into clinical practice will also require mental health providers to comprehend new methods that will empower them to critically evaluate emerging literature and tools. This systematic review synthesizes current findings from AI and its application to OCD, and highlights opportunities for future research that will advance early OCD symptom detection, scalable ERP therapy training, clinical decision support tools, novel therapeutics, computer vision-based approaches, and multimodal biomarker discovery to improve OCD outcomes.

## Methods

### Search Strategy and Selection Criteria

In this systematic review, we searched for peer-reviewed articles on AI, ML, or NLP for OCD. We used three electronic databases, PubMed, Embase, and Scopus, combining three concepts: intervention (e.g., AI, ML, or NLP), condition (OCD), and outcome (e.g., diagnosis, prediction, treatment, and management), without language restrictions. A complete search strategy is in Appendix 1. The first search was conducted in October 2024, and an updated search was done on February 4, 2025.

Studies were included if: (1) it investigated the use of AI, ML, or NLP for OCD; (2) there was a separate outcome for OCD, if OCD was one of the psychiatry disorders in the study; (3) it was a protocol or case study, and the outcome measurement metric was clearly defined, because we aimed to overview the use of AI for OCD broadly, not limiting it to clinical outcomes, sample size, or outcome measurement type. We excluded a study if: (1) it used a biomarker- or physiology-based detection (e.g., electroencephalography [EEG] data) or intervention (e.g., deep brain stimulation [DBS], electroconvulsive therapy [ECT], repetitive transcranial magnetic stimulation [rTMS], transcranial direct current stimulation [tDCS] for OCD) due to the existing literature [15]; (2) it was a review or editorial.

### Data Extraction

Two individuals conducted eligibility evaluation, data extraction, and quality assessment independently (JK and JP). For full-text review and data extraction, a third reviewer resolved conflicts when needed (CIR). We utilized Covidence (Australia) to remove duplicates, screen abstracts, review full articles, and collect data, which were exported to Microsoft Excel (United States). JK created a summary table using the extracted data, and JP validated data import for quality. All coauthors reviewed and confirmed the final tables. We followed the PRISMA guidelines (Appendix 2).

We anticipated significant variability of outcomes and approaches, given that we included studies that investigated AI in OCD broadly, not limiting our review to studies of clinical outcomes only. Hence, we slightly modified the categories of PICO (Population, Intervention, Comparison, Outcomes) when systematically synthesizing the characteristics of included studies to optimally extract all the relevant information. We collected information, including study populations for human subject study/data sources for secondary data analysis, intervention/approach/specific AI or ML models, study aim, study location, sample size, analytic methods, outcomes, and interpretation and future directions.

To assess the quality of evidence, we used a modified version of the Grading of Recommendations Assessment, Development, and Evaluation (GRADE) guidelines for rapid reviews [16]. Certainty of evidence was evaluated as high, moderate, low, or very low, based on the author’s confidence, considering multiple domains that may increase (e.g., dose-response gradient, the magnitude of effect, or minimum confounding factors) or reduce (e.g., limitation of study design, inconsistency in results, imprecision, and publication bias) the certainty of evidence. Two reviewers independently assessed the quality of the evidence (JK and JP). A third reviewer reconciled conflicts between the two (CIR).

## Results

**Fig. 1:**
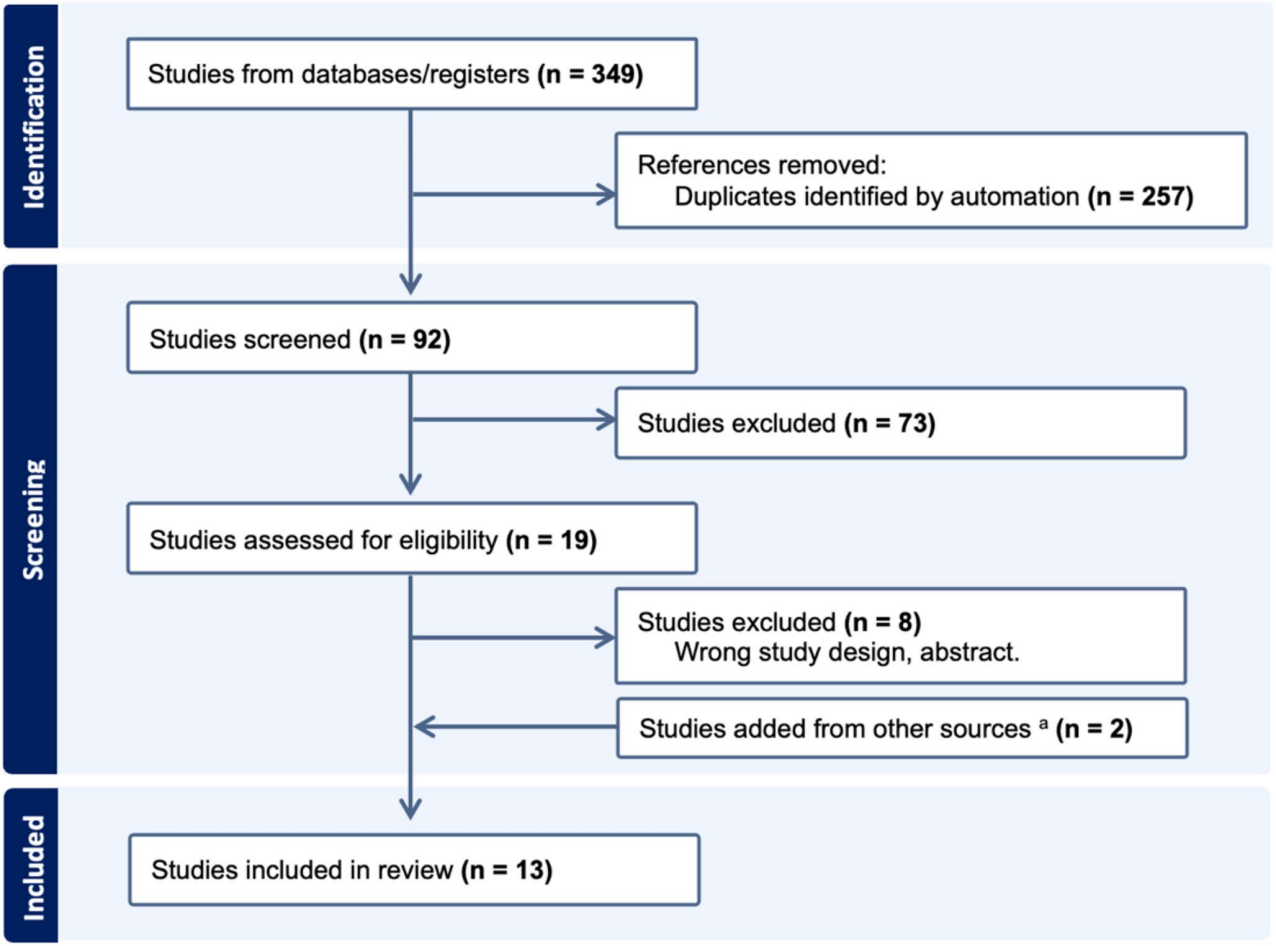
Study flow and selection. ^a^ Other sources: one study was found from generative AI-assisted search (e.g., OpenAI Deep Research, February 2025, OpenAI Inc.) and one study was found by snowball search

Initially, 349 articles were retrieved, and 92 studies were screened for titles and abstracts after excluding duplicates by automation (Fig. 1). We included 19 studies to assess eligibility through full-text review and excluded 8 studies due to wrong intervention (e.g., use of physiology-based detection). By adding 2 studies found from other sources, we had 13 studies in this systematic review.

Table 1 shows the characteristics of the included studies. Most studies were secondary data analyses (84.6%, *n* = 11/13), text data from published articles, social media, a large population database, mobile applications, or audio data from another clinical trial; two studies involved human participants. The language analysis approach was the most common (69.2%, *n* = 9/13), followed by prediction modeling (23.1%, *n* = 3/13), and one framework evaluation (7.7%, *n* = 1/13). The publicly available data set was used most frequently (53.8%, *n* = 7/13), including social media posts, mobile app data, and published articles. Private data sets from the human subject study and LLM-generated data were both 23.1%, 3 studies each. The majority of studies (84.6%, *n* = 11/13) employed quantitative outcomes (e.g., accuracy/F-1 score [*n* = 5], OR [*n* = 2], coherence score [*n* = 1], WEAT [*n* = 1], ANOVA [*n* = 1], mean [*n* = 1]); while two studies used mixed features of qualitative and quantitative measurement (e.g., heuristic clustering, scores for credibility, user experience, user agency, equity and inclusivity, transparency, safety and crisis management). Most were published in the past two years (76.9%, *n* = 10/13, since 2023), and more than one-third of the studies were from the US (38.5%, *n* = 5/13), while 23.1% were from Europe (*n* = 3; UK = 1, Denmark = 1, Russia = 1), and 23.1% were from Asia (*n* = 3; India = 1, China = 1, Saudi Arabia = 1). Two studies used no country-specific data; one conducted in the US used English word-embedding data. Another study conducted in Iran used published articles in English.

**Table 1:** Study characteristics: data source of participants, intervention/approach, and outcomes

	Authors	Year	Study aim	Population/ Data source/ Sample size	Intervention/ Approach/ Models	Outcome measures	Findings/ Conclusion
1	Mil et al. [17]	2024	To assess the psychosocial functioning of OCS/OCD comorbidity in people with schizophrenia, schizoaffective disorder, or bipolar disorder	15,412 subjects from SLaM BRC (01/2007- 12/2016)	Language analysis: NLP software (GATE)	ORs of psychosocial functioning (problems with activities of daily living, living conditions, occupational and recreational activities, and relationships)	Comorbid OCS/OCD was associated with poorer psychosocial functioning in people with schizophrenia, schizoaffective disorder, or bipolar disorder.
2	Khazaneha et al. [18]	2024	To classify OCD medications by relevance and categorize the relationships	Through the published articles with an initial search of 6574	Prediction modeling: DT, chi-square automatic interaction detection (CHAID) algorithm, and linear model	Total link weight index (strength of relationships); EWKM diagram; 5-fold crossfold validation (accuracy, recall, precision, F-1)	ML analysis provided valuable insights into the efficacy of various medications, such as clomipramine, duloxetine, and pindolol, as well as supplements such as folate, in the treatment of OCD.
3	Feusner et al. [19]	2021	To examine obsession symptoms from an OCD mobile app based on their latent semantic relationships in the English word embeddings	7001 unique words representing obsessions from 25,369 individuals	Language analysis: Global Vectors for Word Representation with a domain-specific extension (Mittens) for OCD-specific words	Clustering for subtypes of OCD	The closeness of the overall embedded relationships across clusters and their central convergence on harm suggests that harm to self or others may be an underlying organizing theme across many obsessions.
4	Ruan et al. [20]	2023	To study the behavioral and neural processes of arbitration	A total of 30 OCD patients and 120 HC	Prediction modeling: 2-choice, 3-stage Markov decision-making (reinforcement learning)	Paired-sample *t*-tests (learning strategy analysis); 1-way ANOVA (task performance analysis)	An impaired arbitration mechanism for flexible adaptation to environmental demands in both OCD patients and HC reporting high OCI-R scores.
5	Clemmensen et al. [21]	2022	To test the association between OCD diagnosis and symptom severity on vocal features in children and adolescents	Audio recordings of clinical interviews of those with OCD (*n* = 47) and those without a psychiatric diagnosis (*n* = 17), age 8–17	Language analysis: vad-crdnn-libriparty pretrained model from SpeechBrain	ANOVA (the effect of OCD diagnosis on scores of vocal activation); logistic regression (the effect of OCD severity classifications on the vocal scores)	N/A
6	Srividya et al. [22]	2018	To identify state of mental health through a new framework for behavioral modeling	656 individuals in target group (high school students, college students, and working professionals, age 18–26)	Prediction modeling: logistic regression, Naïve bayes, support vector machines, DT, KNN	Accuracy, precision, recall, and F-1 scores	A framework was developed for determining the state of mental health, which can be used to build prediction models. The ensemble classifiers improved the performance of mental health prediction with 90% accuracy.
7	Plank and Zlomuzica [23]	2024	To extract linguistic markers from social media, which are of indicative of the onset and course of mental disorders	Subreddits of mental health domains (“r/OCD”), 01/01/2021-12/31/2023; For OCD, 102,018 posts filtering from 208,556 members	Language analysis: 512-dimensional semantic space, using GUSE	Coherence scores	Coherence scores were the highest in the HC, followed by r/depression, r/anxiety, and r/OCD (m[*sd*]: 0.268 [0.0003])
8	Kim et al. [24]	2024	To examine the diagnostic accuracy of LLMs compared to clinicians and other mental health professionals using clinical vignettes of OCD.	8 types of OCD vignettes (*n* = 51) and 7 other psychiatry disorders as control vignettes (*n* = 21)	Language analysis: ChatGPT 4, Gemini 1.5, Llama 3	Diagnostic accuracy from zero-shot identification (%)	LLMs were consistently more accurate in identifying OCD vignettes compared to mental health and medical professionals, doctoral students, and clergy members.
9	Brandsen et al. [25]	2024	To investigate the presence of bias in AI language models related to a range of neurodivergent conditions, including autism, ADHD, schizophrenia, and OCD	Words embeddings from 11 AI language models	Language analysis: 11 models: all-distilroberta-v1, sentence-transformers/all-mpnet-base-v2, sentence-transformers/all-MiniLM-L12-v2, Distiluse-base-multilingual-cased-v2, Multi-qa-distilbert-cos-v1, multi-qa-mpnet-base-dot-v1, paraphrase-MiniLM-L3-v2, Paraphrase-albert-small-v2, and Msmarco-distilbert-base-tas-b, GPT2 and OpenAI 0.26.4.	16 biases using Word Embedding Association Test (WEAT) score; Sentence Embedding Ratio Test (SERT) score	Overall high level of bias toward or negative associations with terms related to neurodiversity. While overall levels of bias vary based on the encoder considered, particularly high levels of average bias were found for tests related to slurs, violence, or obsessiveness.
10	Koltcov et al. [26]	2024	To explore the potential of zero-shot classification of LLMs to select and pre-classify texts into topics representing psychiatric disorders	Russian social media/public forums (b17 Russian Psychological Forum; VK Social Network; Russian-language Reddit Network; (OCD: *N* = 5,393)	Language analysis: mDeBERTa-v3-base-xnli-multilingual-nli-2 mil7, multilingual-MiniLMv2-L6-mnli-xnli, distilbert-base-uncased-mnli, DeBERTa-v3-base-mnli-fever-anli	F-1 score	LLM fine-tuning makes a far larger contribution to its quality. Both standard and natural language inference (NLI) modes of fine-tuning increase classification accuracy by more than three times compared to non-fine-tuned training with preliminarily filtered data.
11	Golden and Aboujaoude [27]	2024	To review the history of rating systems used to evaluate AI mental health interventions, to describe the recently introduced Framework for AI Tool Assessment in Mental Health (FAITA-Mental Health), to demonstrate the use of FAITA-Mental Health to OCD Coach	N/A	Framework evaluation: OCD Coach (commercially available GenAI tool for OCD management)	Credibility, user experience, user agency, equity and inclusivity, transparency, safety and crisis management	Most of the framework’s domains and subdomains could be effectively assessed and scored. However, several potential areas of refinement were identified.
12	AI-Haider [28]	2024	To investigate and detect OCD in Arabic tweets	8,711 Arabic posts from Twitter (03/2022-09/2022)	Language analysis: fastText, TF-iDF and ML classifiers (DT, RF, KNN)	Precision, recall, and the F1-score	Efficient word representation approaches combined with recent ML models have shown reasonable progress on text classification tasks.
13	Bernstein [29]	2025	To examine the feasibility and promise of LLMs to generate appropriate exposure suggestions for OCD treatment	AI-generated partial (*n* = 15) or complete (*n* = 55) responses	Language analysis: ChatGPT-4	Appropriateness, specificity, variability, and usefulness	ChatGPT-4-generated hierarchies were appropriate, specific, variable, and useful.

*OCS* obsessive-compulsive symptoms, *OCD* obsessive-compulsive disorder, *GATE* General Architecture for Text Engineering, *SLaM BRC* South London and Maudsley NHS Foundation Trust Biomedical Research Centre, *EWKM* Entropy-weighted k-means, *HoNOS* Nation Outcome Scales, *DT* Decision Trees, *RF* Random Forest, *KNN* K-Nearest Neighbors

Studies that aimed to generate knowledge to enhance OCD treatment or management were the most common (38.5%, *n* = 5/13). These highlighted the importance of treating comorbid psychiatric disorders (e.g., schizophrenia, schizoaffective disorder, or bipolar disorder) or other concomitant illnesses (e.g., hypothyroidism and streptococcal infection); enhanced understanding of subtypes of OCD (e.g., identifying harm to self or others as an underlying central theme across many obsessions) or the role of the arbitration process in OCD symptoms; or explored the ability of large language models (LLMs) to create exposure hierarchies in OCD treatment. An equally common theme was timely OCD detection and diagnosis (38.5%, *n* = 5/13): behavioral modeling-based prediction, linguistic marker-based detection, non-English word-based symptom detection, diagnostic support, or speech-based symptom severity assessment. Lastly, AI tool development was a common topic (23.1%, *n* = 3/13): bias in language models, disorder-specific conversational agent in low-resourced language, or evaluation metric for AI in mental health (Table 2). For the overall certainty of evidence, we started with a presumption of ‘moderate’ certainty and increased our rating to ‘high’ certainty after assessing categories for increasing or reducing certainty of evidence. We increased our certainty rating by one grade due to the large sample sizes of included studies, while no category suggested downgrading of our certainty estimation. Please see the full GRADE assessments in Appendix 3.

**Table 2:** Study characteristics: implications and future directions

**Data set**		
Public data (population data registry, published article, social media post)	53.8% (*n *= 7/13)	
Private data (human subject studies)	23.1% (*n* = 3/13)	
LLM-generated data (exposure suggestions, diagnosis and reasoning, management suggestions)	23.1% (*n* = 3/13)	
**Location of study (country)**		
US	38.5% (*n *= 5/13)	
Europe (UK, Denmark, Russia)	23.1% (*n* = 3/13)	
Asia (India, China, Saudi Arabia)	23.1% (*n* = 3/13)	
Other^a^	15.3% (*n* = 2/13)	
**Topics of study (implications and future directions)**		
***OCD treatment and management***		38.5% (*n* = 5/13)
• Identifying and treating comorbid OCS/OCD among those with schizophrenia, schizoaffective disorder, or bipolar disorder is important [17]		
• Treating concomitant diseases, namely hypothyroidism and streptococcal infection could improve the efficacy of treatment [18]		
• A new conceptual framework with understanding how an apparent multitude of obsessional symptoms are connected could aid exposure-based treatment [19]		
• Understanding the role of the arbitration process in OCD and impairments in instrumental learning may underlie the symptoms of OCD		
• Enabling professional and paraprofessional support to deliver personalized, high-quality ERP for OCD, that can allow patients to use self-help apps more effectively [29]		
***OCD symptom detection and diagnosis***		38.5% (*n* = 5/13)
• Behavioral modeling for other mental illness and different sections of the society [22]		
• Coherence analyses for early detection and prevention from public data could open new avenues for large-scale prevention programs aimed at high-risk populations [23]		
• Assessment of LLMs’ effectiveness for diagnostic assistance and treatment suggestions for psychiatric disorders within mental health and primary settings [24]		
• Vocal sensing-based automated severity assessment and monitor of psychiatric disorder such as OCD is promising [21]		
• Development of a pretrained model designed for mental health is crucial, as it could help identify subtle linguistic cues that indicate different mental health conditions [28]		
***AI tool development for OCD***		23.1% (*n* = 3/13)
• Identifying ways to mitigate social bias, increase the scope of biases, and engage with neurodivergent groups could address these biases [25]		
• New dataset and fine-tuned models allow to develop a conversational agent in low resourced language (e.g., Russian) for disorder-specific mental health counseling [26]		
• Stringent standards to guide AI integration into mental health care effectively and safely to protect users’ rights and welfare are needed [27]		

^a^ Other: Two studies used no country-specific data; one conducted in the US used English word-embedding data. Another study conducted in Iran used published articles in English

*Abbreviations*: *NLP* natural language processing, *ML* machine learning, *LLM* large language model, *US* United States, *UK* United Kingdom, *OCD* obsessive-compulsive disorder, *OCS* obsessive compulsive symptoms, *ERP* exposure and response prevention therapy, *AI* Artificial Intelligence

## Discussion

In this first systematic review of AI in OCD, identifying 13 relevant articles, most studies (84.6%) were secondary data analyses using text data from various sources. The language analysis approach was the most common (69.2%), followed by prediction modeling (23.1%). The public data, including social media posts, mobile app data, and published articles, was most frequently analyzed (53.8%). Nearly 77% of studies were published in the past two years (since 2023), and the certainty of evidence was considered high. We also found that there were mainly three focus areas of AI in OCD. The primary area was enhancing treatment and management, and timely OCD detection (both 38.5%), followed by AI tool development for OCD and mental health (23.1%). After reviewing the salient themes, we explored gaps and opportunities for future research, which we elaborate below.

### Early Detection and Prediction of OCD Symptoms through Automated Analysis of Clinical Encounters and Patient-Generated Text

Findings highlighted that identifying the indications of onset or the course of mental disorders through behavioral modeling or large sets of user-generated data analysis from social media and mobile apps could be promising approaches for timely detection of symptoms. Non-English social media data analysis contributed to creating a knowledge base of OCD symptom detection beyond English speakers, including Russian and Arabic [26]. Transfer learning—where pre-trained language models are fine-tuned on domain-specific data—could be explored further to improve diagnostic accuracy, especially in low-resource settings [28, 30]. LLMs, the AI systems that drive chatbots like ChatGPT and DeepSeek, offer new tools for mental health care. LLMs’ advanced clinical reasoning holds promise in assisting mental health care providers in detection, diagnostic assessments, and a treatment plan [31–34]. Indeed, diagnostic abilities of LLMs for OCD compared to providers was tested by using clinical vignettes (short descriptions of fictional cases that capture OCD symptoms); surprisingly, LLMs outperformed mental and medical health professionals in identifying OCD [24]. Encouragingly, the applications of various proven approaches, including prompts engineering, few-shot learning, or fine-tuning, could even boost LLMs’ capacity to detect OCD symptoms [35, 36]. Another promising aspect of LLMs may include serving as a symptom screener and assisting clinicians in enhancing timely text-based symptom detection from patient-generated clinical data. A recent study demonstrated LLMs’ high accuracy in detecting poor mental health symptoms from secure patient messages [36]. The small open-source model’s exceptional accuracy in text-based symptom detection signals various open opportunities for the efficient use of LLMs in clinical settings. Lastly, vocal-feature-based symptom detection or severity assessment also holds promise as the technology rapidly evolves, and high-performing multimodal and multilingual models keep being introduced, opening the chapter on speech-based diagnosis [21]. Integrating multimodal data (e.g., physiological signals, behavioral markers) could enhance prediction accuracy. This is particularly relevant for developing AI-driven tools to monitor OCD symptom progression or assess treatment efficacy in real time.

### Enhancing ERP Therapy Training Scalability and Clinical Support Tools

As described previously, ERP is a recommended first-line treatment for OCD [1], but it is not widely available [8, 9]. A major reason for this is a shortage of ERP-trained providers [10]. LLM-based tools could be used to support broader implementation of ERP. They can be employed to enable more scalable training approaches that reduce expert trainer time, such as enabling therapists to practice delivering ERP to AI patients while receiving feedback from an AI-powered consultant [37, 38]. LLMs could also be used to develop clinical support tools, such as help therapists efficiently construct optimally tailored, high-quality exposure hierarchies, which are critical to effective care [29]. Likewise, patients could use LLM-based tools to facilitate between-session homework completion, since low homework adherence can compromise treatment effectiveness [39]. All of these potential applications of AI to reinforce evidence-based care for OCD must carefully consider and seek to prevent possible unintended problematic uses, such as patient LLM-based tools supporting and worsening reassurance seeking or ritualizing behaviors. In the shorter term, until safety and effectiveness are well-established, it will be essential to ensure that a provider remains involved in reviewing the interactions [37]. The field is rapidly advancing, and LLM-based patient-facing tools are likely to become widely available. Careful evaluation of factors—including safety, privacy, effectiveness, engagement, equity, and potential for integration into clinical care—will be necessary to ensure ethical and successful implementation into models of clinical care [40].

### AI-augmented Drug Development

As we understand more about the underlying etiology and pathophysiology of complex mental illnesses like OCD, AI technologies will play a critical role in the drug development pipeline to improve efficiency and effectiveness of treatments. As reviewed by Zhang and colleagues [41], AI applications can be used in target identification, drug discovery, preclinical and clinical trials, approval, and post market surveillance. As one example, advances in high-resolution structural information of G protein-coupled receptors (channels that transmit extracellular signals into cells) have led to a greater understanding of the molecular mechanisms of receptor activation, which, in turn, serves as a basis for drug discovery [42]. New technologies such as AlphaFold—an AI system developed by Google’s DeepMind—have had a significant impact on protein structure prediction [43], which, in turn, helps identify new potential drug targets. In addition to greater efficacy, there is also a need to develop OCD pharmacotherapy with fewer side effects. SRIs can cause sexual side effects, weight gain, or other dose-limiting side effects. For individuals who are not helped by first- or second-line OCD pharmacotherapy [44], promising novel OCD therapeutic candidates are being studied in clinical trials, including ketamine (NCT05940324), the ketamine metabolite (2R,6R)-hydroxynorketamine (RR-HNK; NCT06575075), nitrous oxide (NCT03826693), MDMA (NCT05783817), and psilocybin (NCT03356483, NCT03300947, NCT06258031, NCT06299319, NCT05546658, NCT05370911, NCT04882839). However, these can also have side effects, including dissociation or hallucination. With AI-powered structure-based drug design, it is now possible to both improve specific and multiple functions while also minimizing off-target side effects [42]. At the same time, there are unique challenges when balancing multiple or competing objectives, such as druggability (e.g., some AI-identified targets do not have suitable binding sites) and synthesizability (e.g., methods for assessing feasibility may be imprecise), as well as technical challenges and computing power limits [41].

### Deploying Ethical and Regulatory Frameworks

While the application of AI holds tremendous promise for the diagnosis and treatment of OCD, it can also carry significant risks related to uncertainty, misuse, harm, and broader societal implications. Given such high stakes and the scale of AI, it is critical to anticipate ethical concerns, evaluate tools systematically, and understand the potential effects on clinicians and patients prior to deployment. Ethical frameworks offer a structured approach for evaluating novel tools throughout the pipeline of development and implementation to ensure that evidence-based needs are motivating the tools, underlying designs are rigorous, and novel tools are integrated successfully within clinical workflows [40, 45–48]. The Framework for AI Tool Assessment in Mental Health (FAITA-Mental Health) standardizes the evaluation of AI-based mental health interventions, especially generative AI technologies [27, 49]. Building on digital assessment scales that predate the AI revolution, such as One Mind PsyberGuide [50], FAITA-Mental Health adopts a multidimensional approach to evaluate six essential domains: credibility, user experience, user agency, inclusivity, transparency, and crisis management, each rated on a 0–2 scale. This multidimensional approach allows systematic comparisons and adapts to the evolving AI landscape, offering a comprehensive, ethically informed evaluation mechanism. Preliminary application to an AI-powered OCD app demonstrates its utility in clinical practice, research, product development, and regulatory guidance. This type of evaluation is the first step in the development and deployment of AI tools.

These frameworks provide a rubric for assessing whether AI tools are developed in alignment with ethical principles, and help guide decision-making during the development process. While the principles may vary, many are rooted in those outlined in the Belmont report (e.g., beneficence, justice, and non-maleficence), while others emphasize values such as transparency and reproducibility [51]. Despite their necessity and value, frameworks may also leave gaps between principles and concrete prescriptive guidance, suggesting that more work is needed. For example, concepts such as “safe” and “responsible” may be broadly defined and accepted, but defined and applied with wider variability in practice. In addition, an overreliance on principles may risk a disconnect between principles and the actual concerns of stakeholders involved: developers, clinicians, patients, and ethicists [52]. To this end, more work engaging with direct stakeholders and with implementation science research is needed to develop context-specific guidelines for clinicians.

### Bias Mitigation

As AI shapes mental health diagnostics and treatment, ethical considerations and bias mitigation are crucial, including OCD. AI models often inherit biases from training data, leading to disparities in care. Brandsen et al. [25] found high bias levels in language models associating neurodivergence with negative stereotypes. Fairness-aware algorithms, diverse datasets, fairness constraints, ongoing audits, and adversarial debiasing can help mitigate these biases. Future research should explore differential privacy to protect patient data while ensuring unbiased, ethical AI decision-making. Prioritizing ethical transparency and bias reduction is essential for equitable mental health care.

### Future Horizons: Computer Vision

Computer vision (CV), a subfield of AI focused on interpreting images and videos—and more broadly, the perceived world—has seen remarkable advances in recent years. Today, CV systems can understand and analyze human activities and behaviors in healthcare settings and even within the home [53, 54]. This capability opens the door to objective, data-driven methods for analyzing and quantifying behaviors characteristic of OCD. Traditional assessments often rely on subjective self-reports or clinician observations, which can be influenced by subjective assessments and may lack consistency. By employing CV techniques, researchers can capture detailed behavioral data, leading to more precise evaluations and a deeper understanding of OCD.

#### Behavioral assessment

An early study introduced a computer-based behavioral assessment specifically targeting compulsive checking behaviors in OCD patients. In this research, participants engaged in a task simulating real-life scenarios requiring checking, such as ensuring that doors are locked or appliances are turned off. The system recorded metrics like the duration and frequency of checking actions. Findings revealed that individuals with compulsive checking tendencies exhibited significantly longer and more frequent checking behaviors compared to control groups, underscoring the potential of computer-based tools in objectively quantifying OCD symptoms. Additionally, virtual reality (VR) has been integrated with CV to assess OCD symptoms within controlled environments. A VR game was developed to provoke and measure subjective and physiological responses associated with OCD. Participants navigated scenarios designed to elicit common OCD triggers, while their behaviors and physiological responses were monitored. The study found that OCD patients exhibited heightened anxiety levels and engaged in more compulsive behaviors.

In another study, CV tools were used to identify behavioral markers in pediatric OCD patients. In this research, youths with OCD and healthy controls (HC) were video-recorded while performing specific tasks, such as arranging objects and hand washing. The recorded behaviors were then analyzed, revealing significant correlations between certain behavioral patterns and OCD symptom severity. Increased time spent ordering objects and more frequent movements during tasks were associated with higher scores on the Children’s Yale-Brown Obsessive Compulsive Scale (CY-BOCS) ordering/repeating dimension. These findings suggest that video-based behavioral measurements can serve as valid, objective markers for quantifying OCD symptomatology. Another study used CV to cluster different kinds of OCD behaviors and determine the approximate level of anxiety represented by compulsive behavior.

#### Pathophysiological understanding

Beyond behavioral assessments, CV has been integrated with neuroimaging and ML techniques to explore the neurological underpinnings of OCD. By analyzing brain imaging data, researchers have identified patterns of functional connectivity that differentiate OCD patients from HC. For example, studies have reported global hypoconnectivity in certain brain networks and hyperconnectivity in regions like the thalamus among OCD patients. These insights contribute to refining pathophysiological models of OCD and may inform the development of targeted interventions.

#### Interventions

Wearable technologies equipped with CV capabilities are being explored for real-time monitoring and intervention in OCD treatment. Devices such as the “Wrist Angel” utilize biosensors to detect physiological signals associated with OCD symptoms, providing continuous data collection in naturalistic settings. This approach not only enhances the ecological validity of assessments but also holds promise for personalized treatment strategies by offering immediate feedback and tracking treatment progress over time.

The integration of CV into OCD research has contributed to providing objective, quantifiable data on behavioral and neurological aspects of OCD, and therefore holds promise to pave the way for more accurate diagnoses, personalized intervention, better-targeted treatments, and continuous monitoring, ultimately improving patient outcomes.

### Multimodal Biomarker Discovery: AI/ML Approaches in Neuroimaging for OCD

Despite significant advances in neuroimaging, a critical translational gap remains in identifying robust, individualized biomarkers for psychiatric disorders broadly [55], and for OCD in particular [56]. Traditional imaging studies have largely relied on group-level contrasts, which limit their clinical utility for diagnosis and treatment prediction at the individual level. In response, there is growing interest in applying AI and ML techniques to neuroimaging data to provide subject-specific, clinically relevant insights [57–61]. In this section, we briefly review recent AI/ML-based research in OCD biomarker discovery and highlight key gaps and future directions. Specifically, we review research in three major application areas: diagnostic classification, treatment response prediction, and symptom subtyping.

#### Diagnostic Classification

The primary application of AI/ML in OCD has been the identification of neuroimaging-derived biomarkers that distinguish OCD patients from HC. Researchers have used various imaging modalities for this purpose—including structural MRI, functional MRI (fMRI), diffusion tensor imaging (DTI), and EEG—with structural MRI being the most extensively studied. Early studies applying AI/ML approaches to structural MRI data reported high classification accuracies in distinguishing OCD patients from HC; however, these studies typically involved small sample sizes or highly selected subsamples [62]. To address these limitations, recent studies have used larger, more heterogeneous samples, yielding mixed results. For instance, a large-scale structural MRI study by the ENIGMA-OCD consortium—analyzing data from over 2,000 patients and controls—found that classification models could not reliably distinguish OCD patients from HC, especially when tested across independent sites. Interestingly, classification performance improved when stratifying by medication status, suggesting that psychotropic medications significantly impact brain anatomy and contribute to the clinical heterogeneity, thereby complicating biomarker discovery using AI/ML [63]. Similar limitations have been observed in white matter diffusion studies, where AI/ML models again failed to produce robust OCD classifications [64]. A parallel ENIGMA-OCD analysis which examined task-free neuroimaging derived functional connectivity as a potential biomarker similarly reported poor classification accuracy, especially in unmedicated patients. Although some consistent connectivity alterations were observed (e.g., hypoconnectivity in sensorimotor networks and hyperconnectivity involving the thalamus), these did not translate into reliable individual-level markers [65]. One notable gap is the underutilization of task-based neuroimaging in OCD, which may provide more direct access to neural circuits implicated in the disorder. Although task fMRI poses logistical challenges—including higher cost, longer scan times, and reduced patient compliance—recent advances suggest that AI can help overcome these barriers. For instance, deep learning (DL) models have been used to accurately predict task-evoked activation maps from resting-state data [66], offering a scalable route to infer cognitive states in OCD without requiring active task engagement. Additionally, more recent studies suggest that *multimodal* approaches—integrating structural, functional, and diffusion neuroimaging data—show enhanced predictive power over unimodal analyses [67]. Furthermore, newer ML algorithms have been successfully employed in large, diverse datasets, such as the ABCD study to extract robust neuroimaging features associated with OCD-related traits [68]. However, several challenges persist: classification accuracy remains modest, model interpretability is often limited, and the generalizability of findings is constrained by cohort diversity and data harmonization issues. Thus, despite promising developments, AI-driven neuroimaging-based diagnosis for OCD remains elusive.

#### Treatment Response Prediction

Another critical application of AI/ML in OCD is the identification of neuroimaging-derived biomarkers that can accurately predict a patient’s response to specific treatments. Recent studies have employed AI/ML models to analyze baseline (pre-treatment) neuroimaging data, aiming to forecast changes in OCD symptoms following treatment. These studies have successfully identified structural and functional MRI-based biomarkers capable of distinguishing treatment responders from non-responders for first-line therapies, particularly SSRIs and CBT [69–71]. However, these findings require replication in larger, independent samples. In contrast, the predictive performance of neuroimaging biomarkers for invasive treatments, such as DBS, has thus far been limited. Improving the accuracy of treatment-response prediction through neuroimaging-derived biomarkers holds significant clinical promise, as it could spare patients from the current trial-and-error approach. Achieving this goal will likely require integrating multimodal data [72], analyzing larger neuroimaging datasets from treatment studies, and employing more sophisticated AI/ML algorithms.

#### Symptom Subtyping

Given the considerable clinical heterogeneity of OCD, a one-size-fits-all approach may lead to suboptimal outcomes. AI/ML methods have been increasingly utilized to parse this heterogeneity and identify neuroimaging-derived OCD subtypes. For example, one notable study employed an unsupervised AI/ML approach on structural MRI data and identified two distinct OCD subtypes [73, 74]. Similarly, another study applied a comparable unsupervised approach to fMRI data and identified three distinct OCD subtypes [75]. These findings suggest that the broad diagnostic category of OCD may encompass multiple neurobiologically distinct subgroups. Identifying robust and replicable subtypes that remain consistent across imaging modalities—and linking these subtypes to clinically meaningful differences using more sophisticated AI/ML methods—will be critical for advancing personalized diagnostic and treatment strategies in OCD.

Beyond these application areas, AI offers promising solutions to long-standing challenges in neuroimaging, such as noise reduction, correction for head motion artefacts, and improvements in spatial and temporal resolution. Leveraging ensemble learning approaches to integrate structural, functional, and diffusion imaging features could help identify converging, multimodal biomarkers that better capture the complex and multifaceted etiology of OCD. In parallel, the use of explainable AI (XAI) is critical for translating complex AI models into clinically interpretable tools [76, 77], especially in psychiatry, where transparency and trust are paramount [78]. Additional promising directions include integrating mobile or low-cost neuroimaging technologies (e.g., EEG, functional near-infrared spectroscopy [fNIRS]) with real-time AI analytics to enable ecological assessment [1], as well as deploying federated learning frameworks to support multi-site collaborations without compromising patient privacy [79].

In summary, although AI/ML are significantly advancing the frontier of neuroimaging-based biomarker discovery in OCD, substantial challenges remain. Addressing methodological limitations, enhancing data quality, increasing sample sizes and participant diversity, and focusing on interpretability and real-world scalability will be essential for translating these innovations into practical tools for precision psychiatry.

## Limitations

While this systematic review highlights promising applications of AI in OCD, several limitations should be acknowledged. First, the review included only 13 articles, reflecting the early stage of AI research in OCD. This relatively small sample size limits the generalizability of the findings and calls for larger, more diverse studies. Second, most studies relied on secondary data analyses, with very few using prospective data analysis or performing real-world validation. This raises critical questions about clinical applicability of the reported AI approaches. Third, the majority of studies focused primarily on NLP/text analysis, neglecting valuable information from other data modalities such as, but not limited, sensor data, neuroimaging, neuropsychological or behavioral assessment data.

## Conclusion

Innovations in OCD mental health care is an urgent public health need, given OCD typically starts in childhood, leads to lifelong morbidity, and costs the economy $2.1 billion (direct costs) and $6.2 billion (indirect costs such as lost productivity) annually [2, 80]. The advent of AI technologies is poised to drive transformation in the field of mental health and in this review, we highlighted current advances that AI has made in OCD. We also highlight several areas where further investment in AI research and resources will yield meaningful enhancements in OCD mental health care across the clinical care continuum.

## Electronic Supplementary Material

Below is the link to the electronic supplementary material.

Supplementary Material 1

Supplementary Material 2

Supplementary Material 3

## Data Availability

No datasets were generated or analysed during the current study.
