# Supplementary material for: Artificial Intelligence in Obsessive-Compulsive Disorder: A Systematic Review
Source: Curr Treat Options Psychiatry. 2025 Jun 14;12(1):23. doi: 10.1007/s40501-025-00359-8 (PMC12167270; doi:10.1007/s40501-025-00359-8)
Supplement: Supplementary file 1 — Supplementary Material 1 [file 40501_2025_359_MOESM1_ESM.docx]

**Appendix 1. Search strategies**

**PubMed in Title/Abstract (N=263)**

1. **Primary Terms:**
   - "Artificial Intelligence" AND "Obsessive-Compulsive Disorder" (n=36)
   - “Large language model” AND “Obsessive-Compulsive Disorder” (n=3)
   - "AI" AND "OCD" (n=47)
   - "Machine Learning" AND "Obsessive-Compulsive Disorder" (n=98)
   - "Deep Learning" AND "Obsessive-Compulsive Disorder" (n=10)
   - "Neural Networks" AND "Obsessive-Compulsive Disorder" (n=34)
2. **Secondary Terms:**
   - "Clinical Decision Support" AND "OCD" (n=1)
   - "Natural Language Processing" AND "OCD" (n=5)
   - "Digital Health" AND "Obsessive-Compulsive Disorder" (n=29)

**Embase (N=18)**

#1. AI

'Artificial Intelligence':ti,ab,kw OR 'Large Language Model':ti,ab,kw OR 'Natural Language Processing':ti,ab,kw

#2. OCD

'Obsessive Compulsive Disorder':ti,ab,kw

#3. Diagnosis/prediction/treatment/management

'Diagnosis':ti,ab,kw OR 'Prediction':ti,ab,kw OR 'Treatment':ti,ab,kw OR 'Management':ti,ab,kw

#1 AND #2 AND #3 = 18

**Scopus (N=68)**

"Artificial Intelligence" OR "Large Language Model" OR "Natural Language Processing"

"Obsessive Compulsive Disorder"

"Diagnosis" OR "Prediction" OR "Treatment" OR “Management”

#1 AND #2 AND #3 = 68

🡪 A total of 349 was imported to Covidence.
