# Supplementary material for: Artificial Intelligence in Obsessive-Compulsive Disorder: A Systematic Review
Source: Curr Treat Options Psychiatry. 2025 Jun 14;12(1):23. doi: 10.1007/s40501-025-00359-8 (PMC12167270; doi:10.1007/s40501-025-00359-8)
Supplement: Supplementary file 3 — Supplementary Material 3 [file 40501_2025_359_MOESM3_ESM.docx]

**Appendix 3. Grading of Recommendations Assessment, and Evaluation (GRADE) Assessment for the overall quality of evidence**

We started with moderate certainty of evidence because most outcomes seemed to lie on the promising side or within a particular range (e.g., on the better half) in terms of quantitative results (n=11/13), however, two studies reported mixed features of outcomes, combining qualitative and quantitative results. Hence, we did not assign the overall certainty of evidence as high.

**Study design and execution**

No clear limitations of study design and execution were observed. The study design was described in the method section properly in all studies (n=13/13). Of which 84.6% (n=11/13) was secondary data analysis, and all specified the data source properly. Two studies involved human subjects with an observational design, which described target group characteristics at a minimum (e.g. age).

**Inconsistency**

No clear limitations were observed regarding the consistency of outcomes. Although no studies examined the same outcomes, the results of the studies were consistent in the same categories in the systematic review, concluding consistently (e.g. the promise of AI in timely detection and diagnosis, the usefulness of knowledge in enhancing OCD treatment and management, and considerations in the development of AI tools for OCD).

**Indirectness of evidence (PICO and applicability)**

Indirectness was considered low because all the outcomes directly answered our research question (Q. How are AI being used for OCD). Because we aimed to synthesize the use of AI for OCD broadly, not limiting it to clinical outcomes, we have slightly modified PICO like below: Population (study populations for human subject study/data sources for study population for secondary data analysis), Intervention (Intervention/approach/specific AI or ML models), Comparison (we reported comparison if any), and Outcomes (Outcomes). All studies provided relevant information for 4 categories, which we critically appraised the synthesized knowledge in the summary table as a main outcome (Table1).

**Imprecision**

Imprecision is considered low because most studies used an ample sample with more than 500 (69.2%. n=9/13), ranging from 656 to 102,028, which was an essential factor for precision. When the study reported multiple outcomes, those were consistent in between. One study conducted a case study using a newly developed framework for AI Tool Assessment in Mental Health, applying it to a GenAI tool. Under the main 6 domains, subdomain scores were reported with detailed reasons based on the evaluation rubric. Hence, we did not consider this case study as a significant limitation to downgrade precision.

**Publication bias**

Publication bias is not considered significant to downgrade. We did not limit the language of articles during the search to three widely used electronic databases and included a study that used Russian online data in this Systematic Review. The location of the study conducted was varied, not being dominated by one country (e.g., USA=5, UK=1, Denmark=1, Russia=1, India=1, China=1, Saudi Arabia=1). We did not search conference proceedings. Hence, it is possible that we might have missed not indexed conference articles. However, overall publication bias is not considered significant to downgrade as we were able to catch indexed conference abstracts through our search strategy.
